# Utilizing BERTopic Modeling for Concept Discovery in the Domain of Gerotranscendence and Solitude

**DOI:** 10.21203/rs.3.rs-7383440/v1

**Published:** 2025-09-19

**Authors:** B. Damayanthi Jesudas, Finn Wilson, Rachel A. Mavrovich, Sean Kindya, Feng-Yu Yeh, Sam Smith, Jeremy Ravenel, Jie Zheng, Yongqun He, Hollen N. Reischer, Julie C. Bowker, John Beverley, William D. Duncan

**Affiliations:** University of Florida College of Dentistry; University at Buffalo; University at Buffalo; University at Buffalo; University of Michigan Medical School; University of Michigan Medical School; University at Buffalo; University of Michigan Medical School; University of Michigan Medical School; University at Buffalo; University at Buffalo; University at Buffalo; University of Florida College of Dentistry

**Keywords:** BERTopic, ontology development, solitude, gerotranscendence, topic modeling, PHASES Ontology

## Abstract

**Background.:**

Ontology development is a complex, iterative process that traditionally requires extensive collaboration between ontology developers and subject matter experts (SMEs). While effective, this manual approach is time-consuming, labor-intensive, and prone to cognitive bias. To streamline early-stage ontology development and uncover concepts that might be overlooked through manual review alone, we applied automated topic modeling with BERTopic to extract topics, keywords, topic labels, and summaries from *The Handbook of Solitude: Psychological Perspectives on Social Isolation, Social Withdrawal, and Being Alone* and *Gerotranscendence: A Developmental Theory of Positive Aging*. The extracted topic labels were used as candidate concepts for the Promoting Healthy Aging through Semantic Enrichment of Solitude Research (PHASES) Ontology.

**Methods.:**

We implemented and compared two BERTopic pipelines: (1) the default configuration and (2) a custom preprocessing pipeline incorporating part-of-speech filtering and n-gram tuning.

The pipeline is customized to flexibly extract any specified number of topics and keywords based on user-defined parameters. To compare and merge topic modeling outputs across solitude and gerotranscendence, we used semantic embeddings of topic labels, keywords, and summaries from the custom pipeline. Cosine similarity identified semantically matched topic pairs above a set threshold, enabling categorization and integration into a merged conceptual framework that bridges both domains.

**Results.:**

From the solitude corpus, BERTopic generated 244 initial topics, which SME review refined to 32 high-quality topics with the custom pipeline and 46 with the default pipeline. For the gerotranscendence corpus, the pipeline produced 172 initial topics, refined to 33 (custom) and 32 (default) high-quality topics. Across both corpora, BERTopic contributed 90 ontology terms, 52 from the solitude corpus and 38 from the gerotranscendence corpus. Visual evaluations, including keyword score bar charts, hierarchical clustering dendrograms, and BART-generated summaries, revealed that the custom pipeline produced more fine-grained, domain-specific topics, while the default pipeline offered broader thematic coverage and clearer labels. Certain theory-laden concepts, however, required SME interpretive input.

**Conclusions.:**

BERTopic provided an efficient, semi-automated approach for identifying candidate ontology terms from domain literature, supporting both breadth and specificity in concept capture. Integrating semantic similarity analysis across thematic domains revealed conceptual intersections and overlaps, enhancing the semantic foundation of the PHASES Ontology and offering a replicable method for cross-domain ontology development.

## 1. Introduction

Ontology development is often an iterative process that involves close collaboration between ontology developers and subject matter experts (SMEs) to identify and represent domain-relevant terms, including both concepts and relations among them. In a standard workflow, ontology developers work with SMEs to identify high-value terms for a given domain, which are then modeled using ontology techniques. The resulting models are evaluated by SMEs for domain accuracy and reviewed by knowledge engineers, who have training in the formal logics of ontology languages, to identify and correct any modeling errors [1]. Afterwards, the resulting models are evaluated by SMEs. Based on the SME feedback, refinements are then made, and new terms are proposed. Many successful ontologies have been created this way, but there are drawbacks. It is labor intensive, often taking substantial time and effort to generate a core set of concepts. The process may also leave out important terms due to biases of the SMEs and developers. Recent advances in the field of natural language processing (NLP), such as the rise of large language models (LLM), have spurred the investigation of how best to use these technologies to facilitate ontology development and address the limitations of manual approaches. DRAGON-AI, for instance, uses LLMs to assist developers in creating textual and logical components [2]. This method leverages Retrieval-Augmented Generation (RAG) to combine the latent knowledge of LLMs with sources like ontologies. While these tools can be very useful in curating ontology content, SME intervention can yield more thematically coherent and domain specific terminology due to their practical experience.

In this manuscript, we applied recent advances in automated text analysis to address limitations in manual approaches to domain-relevant term identification for ontology development in the Promoting Health Aging through Semantic Enrichment of Solitude Research (PHASES) project. The project pertains to solitude, or the state of being alone and away from others, and gerotranscendence, a developmental shift in perspective toward greater personal coherence and acceptance, interpersonal and cosmic connectedness, and decreased materialism in later life [3]. Interestingly, both constructs are relevant for healthy aging, but have typically been studied separately, within different subdisciplines of psychology and/or health-relevant areas of scholarship (e.g., public health, nursing). However, developing a structured, semantically rich ontology encompassing both psychological constructs could bridge these domains, facilitate integration with clinical terminologies, and support computational analyses aimed at advancing mental health care and quality-of-life research for aging populations [4].

We employed BERTopic [5],an advanced topic modeling technique is used to extract topics and associated keywords from one selected handbook on solitude and one selected book on gerotranscendence, chosen in consultation with SMEs on the PHASES project, largely for their rich and conceptually diverse domain content. Two BERTopic configurations were evaluated: one, a default parameterization and another, a custom preprocessing pipeline incorporating part-of-speech (POS) filtering and n-gram tuning to enhance conceptual specificity. The outputs from both pipelines were subsequently reviewed by SMEs to eliminate non-essential terms and refine the remaining results. We then compared the curated BERTopic derived terms with those obtained through the traditional SME-developer interaction method. To facilitate semantic integration, we implemented a cross corpus semantic comparison pipeline. We proposed a data-driven approach to discovering relevant themes in solitude and gerotranscendence could reveal important concepts for each domain that traditional ontology development workflows might overlook. This approach employed sentence embeddings and cosine similarity to identify thematically related topics between the two corpora. The resulting strongly aligned conceptual pairs were proposed as candidates for higher-level merged concepts, contributing to a more interconnected and semantically coherent ontology structure. Our results support this view, revealing 90 novel topics that had not been previously identified. A limitation of this approach is its reliance on BERTopic modeling, which may introduce biases or omit nuanced concepts depending on parameter setting and corpus characteristics. BERTopic outputs can also include semantically light words, repetitive phrases, and citation-derived author names, particularly in smaller topic clusters. While POS filtering and preprocessing reduce much of this noise, further refinement through lemmatization, deduplication, and domain-specific stopword lists can improve topic interpretability.

## 2. Background Literature Review

The use of LLMs in ontology development is driven by the need for semantically rich, context-aware knowledge representations across a variety of domains. This approach enables the precise generation of domain-specific knowledge from relevant corpora, facilitating the development of complex, semantically interoperable ontologies. For instance, the development of the PHASES ontology within the healthy aging domain includes complex psychological constructs like solitude and gerotranscendence, both of which have recognized relevance to mental health, quality of life, and psychological determinants of health in aging populations. We use topic and language models for ontology population and refinement to explore facets of healthy aging.

A 2023 analysis of 44,343 Weibo, a Chinese microblogging website, using BERTopic, identified four major public concerns about active aging in China post-COVID [6]. However, the exclusive reliance on Sina Weibo data introduced platform bias and excluded less digitally literate elders, underscoring the importance of diverse data sources for representative ontology concept extraction. The current approach lacks coverage of offline interviews, cross-platform validation, and mechanisms to filter non-genuine content, limiting the robustness of findings. Longitudinal topic modeling of 5,610 abstracts on “successful aging” from 1963–2023 revealed the persistent dominance of health and social domains, alongside recent growth in mental health, physical activity, and social participation [7]. While comprehensive, this study was limited by its reliance on abstracts and lack of discipline-specific semantic granularity. An analysis of 63,809 tweets using BERT-based Named Entity Recognition (NER) and BERTopic captured semantically coherent topics on public perceptions of healthy ageing [8]. While offering insights, topics such as ‘frailty’ and ‘elder abuse’ were absent, reflecting differences between tweets and academic discourse, reinforcing the value of curated scholarly corpora for ontology development.

A 2024 study proposed an explainable machine learning-based healthy aging scale built from survey data of 696 Slovenian adults aged 50+, with continuous input from gerontology experts [9]. Explanatory Factor Analysis identified physical, mental, and social health constructs, which experts rated via a custom web annotation tool to create ground truth. Six classifiers were tested, with XGBoost achieving the best performance (AUC = 0.92, F1 = 0.76), and SHapley Additive exPlanations (SHAP) was applied to provide transparent, interpretable predictions for decision support use. However, the method was limited by moderate, single-timepoint self-reported data. In work by Kuspinar et al. [10], NLP was used to identify six key domains—pain, walking, standing, stairs, sleeping, and playing with grandchildren—toward developing a new osteoarthritis-specific preference-based Health-Related Quality of Life (HRQL) index from 102 Canadians with hip or knee osteoarthritis. BERTopic was selected for its ability to efficiently cluster semantically similar responses, reduce researcher bias, and capture nuanced patient concerns. The work also enabled hierarchical topic merging for domain refinement, though limitations related to data completeness, recruitment approach, regional variation, and methodological validation remain.

The application of LLMs in qualitative health research presents both opportunities and challenges, as highlighted by Castellanos et al. [11]. Their work examined how ChatGPT[12, 13] could augment thematic analysis of healthcare forum data alongside Latent Dirichlet Allocation (LDA) [14] topic modeling. ChatGPT contributed depth through subtheme generation and complementary insights which helped uncover hidden structure within broad topics and surface specific facets that human coders might overlook. However, it also carried risks such as overfitting and misclassification of divergent themes. Additional limitations include an exclusive focus on a single nurse forum, reliance on GPT-3.5/4 models, and the use of basic prompting strategies. Li et al. [15] systematically reviewed 30 studies on LLM applications in ontology engineering, using Kitchenham’s methodology [16] to analyze tasks, models, datasets, and evaluation methods. The review finds most work focuses on implementation tasks such as conceptualization, encoding, matching, and evaluation, with fewer studies on requirements specification, publication, and maintenance. LLMs serve as ontology engineers, domain experts, and evaluators, using inputs from text to Web Ontology Language (OWL) [17] ontologies to generate outputs like competency questions, SPARQL [18] queries, axioms, and documentation. Strengths include comprehensive task mapping, model diversity, and openly shared datasets. Limitations involve inconsistent task definitions, lack of standard benchmarks, limited reproducibility, and under exploration of later Ontology Engineering (OE) phases. Nayyeri et al. [19] present RIGOR, a Retrieval-Augmented Iterative Generation framework that uses LLMs to transform relational database schemas into rich OWL ontologies with minimal human input. Context from the schema, documentation, core ontology, and external ontologies guides LLM-generated ontology fragments, which are refined by a Judge-LLM before integration. Applied to medical databases, RIGOR produced consistent, semantically aligned, standards-compliant ontologies, outperforming baselines in accuracy, completeness, and clarity. The method reduces manual effort and highlights the synergy between ontology engineering and LLMs, though occasional issues such as ambiguous property definitions, incomplete equivalence links, and inconsistent class typing remain.

### 2.1 Topic Modeling for Ontology Development

Topic modeling, a widely used statistical technique, can be used in ontology development to uncover latent thematic structures within large collections of documents by identifying clusters of related words that represent topics [20]. These themes enable concept identification, thematic structuring, domain representation and ontology evolution, thereby facilitating the semi-automated development of a domain specific ontology. Topic modeling is a powerful tool for enriching ontologies with formal terminology by capturing core concepts from the corpora that may be overlooked in manual approaches. Fuellen et al. [21] highlight the use of text mining and automated information extraction to populate aging-related databases, capturing explicit facts and infers implicit knowledge from literature. This approach supports ontology development by identifying and grouping domain-specific concepts and hypothesizing relationships that can be formalized into ontology structures. Such workflows parallel the role of topic modeling, which could further enhance the discovery and clustering of concepts prior to their integration into ontologies. Incorporating these methods enables more comprehensive, up-to-date, and semantically rich ontologies for aging research. Traditional topic modeling methods include Latent Dirichlet Allocation (LDA), a generative probabilistic model that represents each document as a mixture of latent topics, where each topic is characterized by a probability distribution over words; Non-negative Matrix Factorization (NMF), an unsupervised learning algorithm that extracts meaningful parts-based representations by decomposing data into additive, nonnegative components; and Latent Semantic Analysis (LSA) [22], which uncovers hidden semantic relationships by reducing term-document data into a similar set of meaningful factors. Egger et al. [23] compared LDA, Non-negative Matrix Factorization (NMF) [24], Top2Vec [25], and BERTopic in the analysis of large-scale social media datasets, demonstrating BERTopic’s superior topic coherence and contextual relevance when applied to informal language sources. Their findings underscore the importance of embedding-based topic modeling approaches for enhancing interpretability in noisy or unstructured text. More recently, advances in deep learning and embedding techniques have led to neural topic models such as Neural LDA [26] and approaches that combine contextual embeddings with clustering algorithms, such as BERTopic and Top2Vec.

In these methods, pretrained language models are employed to generate dense semantic embeddings that capture the contextual relationships between terms and documents. The high-dimensional embeddings are subsequently projected into a lower dimensional space using techniques such as Uniform Manifold Approximation and Projection (UMAP) [27], facilitating more efficient structure discovery. Clustering is then performed using algorithms such as Hierarchical Density-Based Spatial Clustering of Applications with Noise (HDBSCAN) [28, 29] to identify coherent and contextually informed topic groupings.

### 2.2 Applications of BERTopic in Health and Aging Research

BERTopic is an advanced topic modeling technique that integrates transformer-based contextual embeddings with class-based Term Frequency - Inverse Document Frequency (c-TF-IDF) [30] to identify dense, semantically coherent clusters of documents. By leveraging pretrained language models to generate high-quality sentence embeddings, BERTopic effectively captures contextual meaning beyond traditional bag-of-words representations. The c-TF-IDF weighting scheme then enhances topic interpretability by highlighting the most representative keywords within each cluster. BERTopic’s key strength lies in its flexibility and extensibility, supporting a variety of topic modeling techniques tailored to diverse research needs. These include dynamic topic modeling, which tracks topic evolution over time; hierarchical topic modeling, which uncovers topic-subtopic relationships; multimodal topic modeling, which integrates multiple data modalities; and semi-supervised and guided (or seeded) topic modeling, which steer the model toward specific thematic areas by incorporating user defined seed topics. Guided topic modeling is useful for domain-specific applications where prior knowledge can focus the discovery process on relevant concepts.

BERTopic has been increasingly applied in health and aging contexts, often in combination with other NLP and machine learning techniques to improve topic interpretability and support decision-making. An analysis of therapist speech from YouTube transcripts used BERTopic together with LLM-based summaries, KeyBERT [31] keyword extraction, and expert-guided refinement to uncover thematic patterns in psychotherapy communication [32]. While GPT-assisted labeling enhanced interpretability, the dataset was small and imbalanced, and omission of contextual cues limited clinical representativeness.

BERTopic has also been used in clinical psychotherapy analysis, where 552 transcripts from 124 patients were modeled separately for therapist and patient speech, yielding 250 topics each [33]. These topics were then applied in machine learning models to predict symptom severity and therapeutic alliance, with model explainability provided via SHAP [34, 35]. Despite its breadth, the study faced limitations in hyperparameter tuning, topic redundancy, and predictive accuracy for alliance outcomes. A PubMed-indexed study by Chiu et al. [36] developed a BERTopic-LSTM hybrid model to predict 30-day ICU readmissions using unstructured discharge summaries from the MIMIC-III [37] database. By transforming clinical notes into topic vectors and combining them with sequence modeling, the approach achieved Area Under the Receiver Operating Characteristic (AUROC) values around 0.80, demonstrating BERTopic’s potential in enhancing predictive modeling from clinical text. Another PubMed-listed investigation by Wu et al. [38] applied BERTopic to MIMIC-III ICU records of 6,600 heart failure patients, integrating unstructured topic features from clinical notes with structured EHR data in a hybrid model. This integration improved mortality prediction accuracy compared to structured-only models, illustrating the complementary role of topic modeling in critical care decision support. In a policy analysis context, Li et al. [39] applied BERTopic to 436 public medical policy documents under China’s Healthy China Strategy, extracting 27 themes related to elderly services, infectious disease planning, health education, and safety regulation. This work demonstrates BERTopic’s applicability in synthesizing large-scale, text-based policy corpora with direct implications for aging populations.

In biomedical literature mining, BERTopic and LDA were applied to 1,837 PubMed abstracts on opioid-related cardiovascular risks in women [40]. While BERTopic demonstrated advantages in clinical text clustering when paired with LLMs, only a single topic from each model was manually reviewed, reducing the robustness of comparisons. Most recently, Chung et al. [41] used dynamic BERTopic with BERT-based NLP to analyze 1,332 stress-related text messages from older adults collected through a mobile health app. The model identified evolving topic clusters such as family stress, financial strain, and health issues providing longitudinal insight into mental health risk factors and supporting early detection of depression in aging populations. This dynamic approach illustrates BERTopic’s adaptability to continuous, time-sensitive data streams in public health monitoring.

### 2.3. Synthesis of Gaps and Relevance to the Present Study

The limitations observed across prior studies, such as small or biased datasets, limited domain coverage, lack of methodological validation, insfficient interpretive depth for theory-laden concepts, and restricted integration between topic modeling outputs and ontology engineering, are partly addressed in our approach. By applying BERTopic to two complementary, domain-specific scholarly books rather than social media or abstract-only datasets, we mitigate platform bias and ensure richer conceptual coverage. The dual-pipeline strategy (custom preprocessing vs. default BERTopic) enables comparison between fine-grained, domain-specific topics and broader thematic patterns, directly addressing the need for methodological evaluation. SME-guided refinement counters automated labeling bias and ensures interpretive accuracy for complex theoretical constructs. Our semantic-embedding-based topic matching bridges thematic domains, solitude and gerotranscendence, supporting ontology integration, an area often underexplored in earlier work. Remaining gaps to address in future work include systematic benchmarking of pipeline performance, expansion to additional aging-related corpora for broader generalizability, and incorporation of iterative SME-LLM collaboration to further streamline ontology concept identification and validation. To operationalize these objectives, the following section details the methodological framework for applying BERTopic pipelines, semantic similarity matching, and expert-guided refinement in the development of the PHASES ontology.

## 3. Methods

The PHASES ontology development involves adding and curating concepts through several stages of iteration and refinement. The concepts are generated based on inputs, including discussions with Subject Matter Experts (SMEs), competency questions (CQ), and insights that emerge from discussions around the CQs. The CQs are natural language questions used in ontology engineering to define the scope, requirements, and intended use of a knowledge model. They capture the kinds of queries the ontology should be able to answer, ensuring relevant concepts and relationships are included, and serve as a validation tool to test whether the completed ontology meets its design objectives [42, 43]. These discussions, involving both SMEs and ontologists, highlight the complex and labor-intensive process in manual ontology development. To address this issue, we propose using NLP techniques, specifically BERTopic modeling, to automate the process of interpretable topic generation from large text corpora which can be identified as ontological concepts. These automatically extracted topics are then reviewed by the SMEs to eliminate any irrelevant terms and ensure domain completeness, resulting in a refined set of concepts for the ontology. By combining BERTopic with SME intervention, the process helps identify overlooked but relevant concepts, minimize bias in topic selection, and maintain comprehensive domain coverage while still reducing some manual effort and supporting a data-driven approach in the early stages of ontology development.

### 3.1 Corpus Selection

To support the development of the PHASES ontology, including both the Solitude Ontology and the Gerotranscendence Ontology, we selected two fundamental books that comprehensively represent the theoretical themes of their respective domains: The Handbook of Solitude: Psychological Perspectives on Social Isolation, Social Withdrawal, and Being Alone [44] and Gerotranscendence: A Developmental Theory of Positive Aging [3]. As comprehensive academic sources, these provide curated overviews and give insights of the key constructs of their respective domains. This allows conceptually rich and thematically coherent topics serving as a foundation for early-stage ontology development. These books enable the extraction of semantically meaningful topics, keywords, and summaries through the application of BERTopic modeling.

### 3.2 Overview of BERTopic Pipeline and Configuration

**Figure 1:**
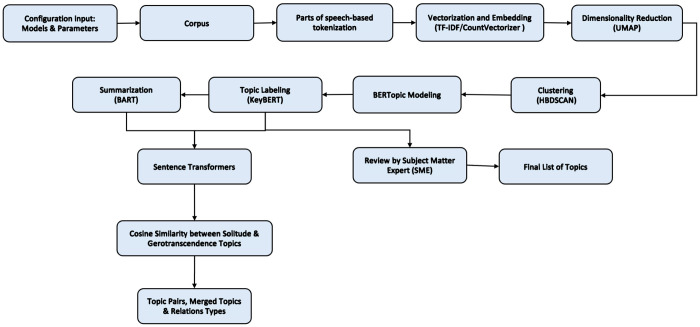
Schematic representation of the methodology.

Figure 1 illustrates the BERTopic pipeline with the modules used in the methodology. A yaml configuration file is used to define and customize the models and parameter values as shown in Appendix A, for each module, enabling flexible and reproducible customization that provides control over the text processing and modeling steps without modifying the underlying code. The key configuration parameters are en_core_web_sm [45], a lightweight and fast language model used in English NLP tasks within the spaCy [45] library. Term Frequency - Inverse Document Frequency (TF-IDF) [46], [47] is used for vectorization along with settings for stopword removal and n-gram range, unigrams, bigrams and trigrams. The all-MiniLM-L6-v2 [48] model, a compact variant of BERT with 6 transformer encoder layers, is used with SentenceTransformer [49] and KeyBERT. The all-MiniLM-L6-v2 model is a compact variant of BERT consisting of 6 transformer encoder layers and is used for embedding sentences, semantic tasks such as sentence similarity, and for extracting keywords from the text to label topics. For dimensionality reduction, UMAP is used. It reduces the high-dimensional BERT embeddings while preserving data structure, which helps in enhancing clustering by revealing patterns and reducing noise. HDBSCAN then identifies dense clusters, handling varied shapes and labeling outliers as noise. After clustering, BERTopic modeling is applied to generate coherent topic representations. These topics are further refined through topic labeling using KeyBERT, and finally Bidirectional and Auto-Regressive Transformer (BART) [50] summarization is used to generate concise and readable descriptions of each topic. The resulting BERTopic results containing the topic labels, keywords, and summaries from the solitude and gerotranscendence domains are used to find the overlapping topic labels from solitude and gerotranscendence. These are embedded using all-MiniLM-L6-v2 to capture semantic meaning. Pairwise cosine similarity is commuted to identify related and strongly aligned topic pairs.

We implemented two versions of the topic modeling pipeline: one using the default configuration of BERTopic and another incorporating customized preprocessing. The default setup relies on a unigram-based CountVectorizer and standard stop word removal. In contrast, the customized pipeline improves domain relevance and topic coherence by applying part-of-speech (POS) [51, 52], filtering and n-gram tuning. The preprocessing process begins by extracting non-empty paragraphs as individual documents. In the customized pipeline, each document undergoes processing with the spaCy library to retain only specific POS categories such as nouns, verbs, and adjectives which are more semantically relevant for topic modeling. The resulting filtered token set is then used to construct a feature matrix through a TF-IDF vectorizer, configured to include n-grams ranging from unigrams to trigrams (1–3 grams). This allows the model to capture both single words and multi-word expressions enhancing the model’s contextual understanding and overall topic quality. POS filtering helps eliminate irrelevant words, enriching the vocabulary representation and increasing the interpretability of the resulting topics. In contrast, the default BERTopic pipeline uses an internal CountVectorizer without any POS filtering or n-gram customization, relying on unigrams and general-purpose stop word removal. While this approach simplifies the pipeline, it can include irrelevant tokens or miss domain-specific terms, which may lead to broader, less coherent topics. Nonetheless, the default configuration remains effective for quick prototyping and general-purpose text analysis, where fine-grained control is less crucial.

Simultaneously, each document is embedded using the all-MiniLM-L6-v2 sentence transformer model, which converts each paragraph into a dense vector representation. The model calculates semantic similarity between sentences by transforming each one into an embedding and comparing the embeddings for similarity. These embeddings are the core input for UMAP, a dimensionality reduction technique that preserves semantic proximity while reducing the vector dimensions. A min_dist value of 0.1 results in denser clusters, and cosine similarity maintains semantic closeness of documents, ensuring that semantically similar documents are grouped together. HDBSCAN then utilizes the UMAP reduced vectors to cluster semantically similar documents, with each cluster representing a distinct topic. Outliers, documents that do not fit into any cluster, are assigned a topic ID of −1, marking them as noise. HDBSCAN outputs both the topic IDs assigned to each document and the probability of each document’s membership in its assigned topic. These outputs are passed to the BERTopic, which interprets the topics based on the topic-document assignments. By default, BERTopic applies c-TF-IDF to aggregate documents within each topic and extract the most important keywords. Next, KeyBERT generates topic labels by identifying the representative document within each topic, providing a concise label that enhances understanding of the topic’s content. The final step involves summarization. Once topic labels and keywords are generated, documents assigned to each topic are aggregated and passed to a pre-trained BART model. This model produces a brief and coherent summary of each topic, offering a concise overview of the topic and the documents within it. The outcome is a comprehensive set of interpretable outputs, including topics, associated keywords, topic labels, topic assignments, automated summaries, and interactive visualizations. These outputs facilitate an effective analysis of the input documents with minimal manual intervention.

To explore conceptual overlaps between topics related to solitude and gerotranscendence, we developed a semantic comparison and visualization pipeline. The outputs from the BERTopic modeling analysis of both datasets, provided as structured .txt files, were parsed to extract topic identifiers, labels, keywords, and summaries. These topic labels, keywords, and summaries were then combined into full textual descriptions, which were encoded into high-dimensional semantic embeddings using the all-MiniLM-L6-v2 model from the SentenceTransformers library. Pairwise cosine similarity scores were computed between topics from the two datasets. A similarity threshold of 0.6 was set to identify conceptually related topic pairs, while a higher threshold of 0.75 indicated strong semantic alignment. Using the all-MiniLM-L6-v2 model, unrelated text pairs typically score between 0.15 and 0.35 in cosine similarity, while related pairs fall around 0.50 to 0.70, and near-paraphrases exceed 0.80. In this study, each topic combines its label, keywords, and summary, which increases similarity scores due to both lexical and contextual overlap. A threshold of 0.60 was selected to filter out chance matches while retaining conceptually related topics across corpora, ensuring enough pairs for meaningful relation extraction. Pairs scoring above 0.75 indicate strong alignment across all fields, distinguishing nearly equivalent concepts from merely related ones. This two-level approach supports both broad exploration and precise identfication of high confidence matches. Matched pairs were then categorized and stored along with their similarity scores, as well as suggested merged concept labels created by combining the original topic labels. To facilitate the interpretation of these results, an interactive bipartite graph was created using NetworkX and Plotly. Topics related to solitude were positioned on the left and colored blue, while topics related to gerotranscendence were placed on the right in green. Edges between nodes reflected the strength of the semantic relationships, with red indicating strong matches and orange denoting related topics. This visualization approach enabled a systematic and interpretable comparison of thematically similar topics across the two analyses and supported the identfication of higher-level conceptual linkages.

## 4. Results

From The Handbook of Solitude, a total of 1866 paragraphs were extracted and treated as individual documents. These were analyzed using both a customized BERTopic preprocessing pipeline and the default BERTopic implementation. The pipeline is customized to flexibly extract any specified number of topics and keywords based on user-defined parameters. This analysis yielded 244 topics, each represented by five keywords, resulting in a combined total of 1220 keywords. Following the removal of duplicate entries and expert review by SMEs to remove irrelevant topics, 32 high-quality topics were retained from the default pipeline and 46 from the customized model, capturing both overlapping and distinct thematic structures. The same dual-pipeline approach was applied to the Gerotranscendence volume, with paragraphs similarly segmented and analyzed. A total of 1249 paragraphs were identified, and this process produced 172 topics, again represented by five keywords each, totaling 860 keywords. After duplicate removal and SME evaluation, 32 high-quality topics were retained from the default pipeline and 33 from the customized model. For the PHASES ontology, after filtering the duplicates from both the pipelines, the BERTopic pipelines contributed a total of 90 terms, 52 from the solitude corpus and 38 from the gerotranscendence corpus

### 4.1 Comparative Analysis of Default and Custom BERTopic Pipelines

**Figure 2:**
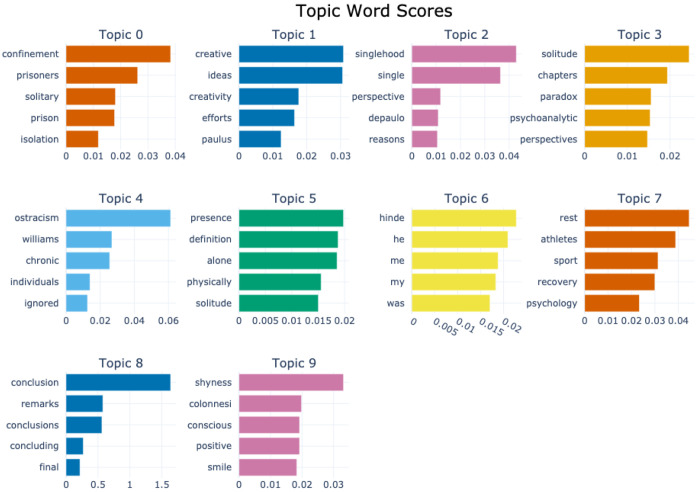
Topic-word score bar chart generated by the default BERTopic model for the solitude corpus.

**Figure 3:**
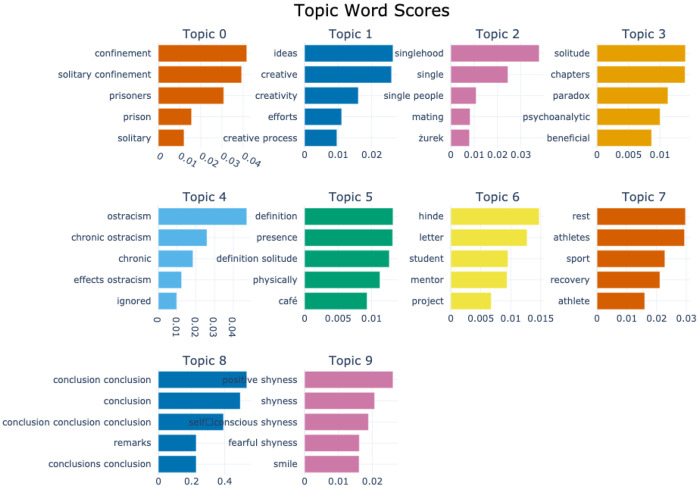
Topic-word score bar chart generated by the custom-preprocessed BERTopic model for the solitude corpus.

Figure 2 and Fig. 3 display topic bar charts from the default and custom preprocessed BERTopic pipelines for the solitude corpus, respectively. Each chart shows the top five keywords for each of the ten dominant topics, with horizontal bars representing each word’s relevance within its topic cluster. Each vertical segment portrays a distinct topic, capturing thematic patterns from the corpora centered on solitude and related constructs. The default pipeline captures a broad range of solitude related themes. For example, topic 0 combines terms like confinement, prisoners, solitary, and isolation, reflecting enforced solitude in carceral or institutional contexts. Topic 1 focuses on creativity, ideas, and efforts, suggesting solitude as a space for cognitive and artistic productivity. Topic 2, with keywords like singlehood and perspective, highlights individual identity and lifestyle aspects related to solitude. Topic 3, with keywords like solitude, chapters, paradox, and psychoanalytic, suggests more abstract or interpretive discourses, possibly literary or theoretical. Some emphasize conceptual or autobiographical reflections, and others center on recovery, affective states, or contain residual academic phrasing.

With custom preprocessing, these patterns become more thematically coherent and domain specific. For instance, Topic 0 is expressed with greater lexical precision emphasizing confinement, solitary confinement, and prisoners. Thematic clarity in topic 1 is enhanced through conceptually aligned keywords such as ideas, creative, creativity, efforts, and creative process. Topic 2 captures relational detachment through relationship terms like singlehood, single, single people, mating. Other topics related to psychological exclusion like ostracism, isolation and psychological traits such as shyness also emerge with more distinct separation from the broader thematic space. Overall, the custom preprocessing improves topic granularity, capturing diverse affective and cognitive facets of solitude. While the default model captures meaningful groupings, its output includes more generic or repetitive terms and lacks domain-sensitive precision. Nevertheless, it remains a useful baseline for exploratory thematic analysis in solitude research.

The substantial overlap between the default and custom BERTopic outputs occurs because both are applied to the same corpus and the use of the same core modeling process, so distinctive terms such as confinement, prisoners, and solitary remain consistent across configurations. Differences arise from how each pipeline tokenizes, filters, and vectorizes text. In the default pipeline, the absence of POS filtering allowed semantically light words, pronouns (e.g., “he”, “me”, “my”, “was”), and author surnames (e.g., “williams”, “hinde”, “zurek”) to appear prominently, often at the expense of thematic clarity. The custom pipeline applies POS filtering, domain stopword removal, and n-gram extraction, producing richer multi-word expressions and removing most low-value tokens, which generally improves interpretability. However, it can introduce visible repetition such as “conclusion conclusion”, conclusion, and “conclusion conclusion conclusion” when bigram and trigram generation interact with c-TF-IDF scoring in small clusters. In these cases, limited vocabulary inflates the relative score of repeated n-grams from headings or summaries, causing duplicates to dominate the top keywords. Both pipelines also retain author names from citation-heavy sections, especially in small topics where frequent terms have greater weight. While the custom pipeline yields more coherent and domain-relevant keywords, targeted lemmatization, n-gram de-duplication, and expanded domain-specfic stopword lists would further improve topic interpretability.

### 4.1 BART-Generated Summaries Providing Interpretive Context for Solitude Topics

Tables 1 and 2 compile the BART-generated summaries for each topic from the default and custom preprocessed pipelines for solitude corpus respectively. These summaries integrate top keywords, topic labels, and contextual interpretations. In the default pipeline model, the summaries contextualize these topics within real-world psychological, institutional, and social frameworks. They highlight solitude’s dual nature—from its harmful expression in solitary confinement to its generative potential for creativity—while also addressing nuanced domains like singlehood, ostracism, and definitional ambiguity. These summaries enrich the interpretability of the keyword-based outputs, although the default model sometimes produces broader, less domain-specific themes. The custom-preprocessed model brings forward more fine-grained and conceptually precise solitude themes. These include intersections with aging, mindfulness, shyness subtypes, hikikomori, behavioral inhibition, and cognitive benfits, alongside familiar domains such as creativity and recovery. Unlike the broader narratives in the default pipeline, these summaries surface nuanced psychological and developmental contexts, revealing solitude’s multifaceted presence across life stages and experiences.

#### 4.1.3 Topic Bar Charts Depicting Keyword Relevance for Gerotranscendence

**Figure 4:**
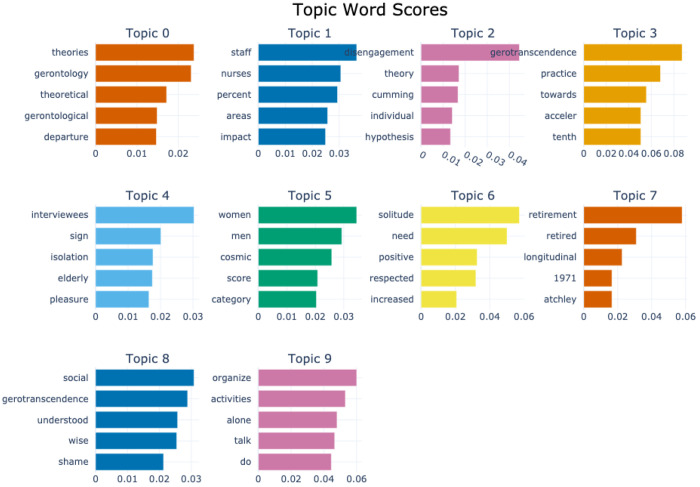
Topic-word score bar chart generated by the default BERTopic model for the gerotranscendence corpus.

**Figure 5:**
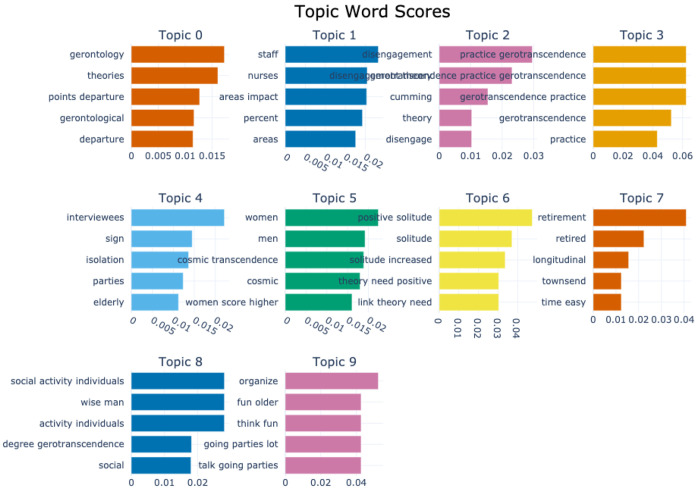
Topic-word score bar chart generated by the custom-preprocessed BERTopic model for the gerotranscendence corpus.

Figures 4 and 5 display topic bar charts from the default and custom-preprocessed BERTopic pipelines for gerotranscendence corpus respectively.

The default pipeline captures a broad range of themes associated with gerotranscendence and aging. Topic 0 features academic and theoretical terms like theories, gerontology, and theoretical, suggesting a general scholarly discourse around. Topic 1 includes staff, nurses, and percent, which point toward empirical or statistical discussions related to the healthcare workforce. Topic 2 is built around the term disengagement, along with theory and cumming, indicating references to the disengagement theory of aging. Topic 3 is dominated by gerotranscendence and related terms like practice, acceler, and tenth, pointing to the conceptual framing and possible empirical measurements of gerotranscendence. Topics 4 to 9 highlight experiential and psychosocial aspects of aging, such as qualitative experiences, gender and existential themes, wellbeing, life transitions, and autonomy. Together, they portray aging as a complex process integrating theory, experience, and identity.

With custom preprocessing, these patterns become more thematically coherent and domain specific. For instance, topic 0 emphasizes theoretical framing with keywords such as gerontology, theories, and points departure, reinforcing an academic inquiry focus. Topic 1 links workforce roles and theoretical perspectives combining staff, nurses, and disengagement. Topics 2 and 3 are both centered on gerotranscendence and its associated practices, with repeated emphasis on its conceptual and practical dimensions, suggesting that this concept appears in multiple discourse contexts within the corpus. The remaining topics span themes of social isolation and qualitative accounts of aging, gendered dimensions of gerotranscendence, psychological benefits of solitude in later life, life transitions and longitudinal perspective, social engagement and philosophical shifts, and lifestyle-oriented narratives in older adulthood.

The differences between the default and custom BERTopic pipelines are largely due to how each pipeline handles preprocessing and vectorization. In the default version, the absence of POS filtering and custom n-gram control allows fragmented tokens, years, and author names such as “acceler”, “tenth”, “1971”, “atchley”, and “cumming” to surface as top keywords, often originating from citations or hyphenated words in the source text. Topics are dominated by single words, which can dilute thematic focus, though this default approach occasionally preserves rare but potentially useful words that heavier filtering might remove. In contrast, the custom pipeline applies POS filtering, domain stopword removal, and n-gram extraction, producing richer multi-word expressions like “cosmic transcendence,” “points departure,” and “positive solitude,” and removing most low-value tokens. This results in more coherent topics, making the output far more interpretable for domain-specific research. However, because bigram and trigram generation is not followed by a de-duplication step, semantically identical phrases such as “practice gerotranscendence” and “gerotranscendence practice” or “social activity individuals” and “activity individuals” can appear together in the same topic. This happens more often in small clusters, as there aren’t many distinct phrases and so both phrases occur frequently enough to get top scores. The c-TF-IDF calculation does not recognize them as duplicates, it just sees two different but frequent phrases and ranks them highly.

Overall, the custom approach is superior for interpretability and thematic precision, while the default is better for broad, exploratory coverage that risks more noise. To make the custom pipeline even cleaner, adding a de-duplication step after vectorization, filtering out of author names and citation patterns, and merging semantically identical n-grams would ensure that each topic’s top terms are both unique and highly informative.

#### 4.1.4 BART-Generated Summaries Providing Interpretive Context for Gerotranscendence Topics

Tables 3 and 4 display BART summarizations for the default and custom-preprocessed models for the gerotranscendence corpus. These summaries present gerotranscendence as a multidimensional development process involving shifts in meaning, perspective, and selfhood in later life. These summaries distill complex themes into clear insights on how individuals transcend ego boundaries, find peace, and reinterpret aging as they grow older. Perspectives from care staff, gender-related nuances, and empirical links to mental health and purpose further enrich the interpretive landscape. The custom preprocessing pipeline, topics are highly focused and conceptually rich topics, emphasizing psychological, spiritual, and developmental aspects of gerotranscendence. This approach enhances topic coherence and separation, allowing clearer distinctions between theoretical constructs, lived experiences, and caregiving contexts. The alignment between the topics and their BART summaries, reinforcing key insights about meaning making and life integration. Overall, custom preprocessing yields a deeper, more structured understanding of gerotranscendence.

Both pipelines perform well but serve different purposes. The custom preprocessing pipeline yields fine-grained, domain-specific insights that are especially valuable for detailed interpretive work, though it may introduce some term redundancy. The default BERTopic pipeline, by contrast, provides a broader thematic overview with cleaner and often more distinct topic labels, making it useful for exploratory analysis. Both approaches rely on c-TF-IDF for generating topic representations, but the custom preprocessing incorporates additional preprocessing steps to improve the quality of the input features before c-TF-IDF is applied, often resulting in more domain-relevant keywords. While the default setup processes raw text with minimal preprocessing, producing broader, more general topic labels, the custom approach enhances specificity and relevance through targeted feature selection. Ultimately, the custom pipeline excels in precision and thematic depth, whereas the default offers breadth, clarity, and reduced redundancy risk.

#### 4.2 SME validation of the topics

**Figure 6:**
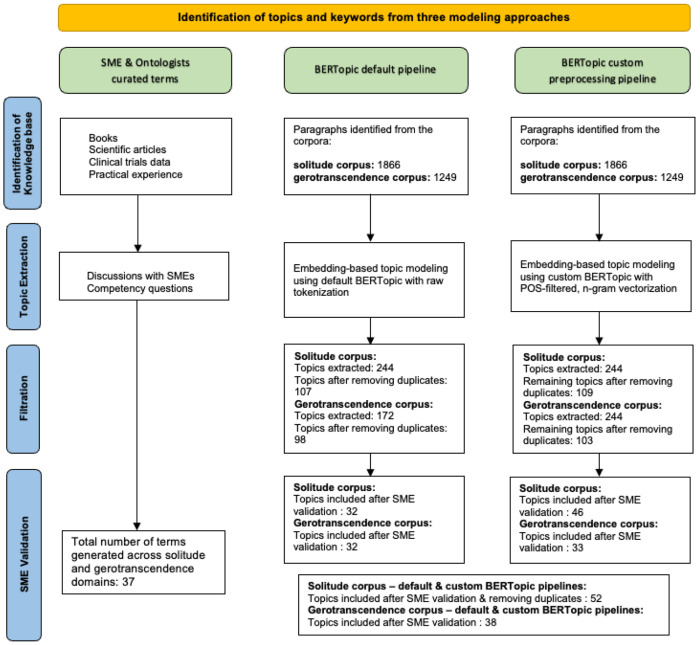
Flow diagram showing extraction, filtering, SME validation, and final inclusion of topics from three modeling approaches

Figure 6 shows the flow diagram of the three modeling approaches, SME curated terms, topics extracted from BERTopic default pipeline, topics extracted from custom preprocessing pipeline, highlighting the steps from initial knowledge base identification to final SME validated topic inclusion. The diagram illustrates the input sizes, topic counts before and after filtration, and the number of topics retained following SME validation.

In the first stage, identification of the knowledge base, relevant content for SME curated terms is sourced from books, scientific articles, clinical trial data, and the practical expertise of subject matter experts. For the BERTopic workflows, the knowledge for topic extraction is sourced from the handbook of solitude and gerotranscendence books. The BERTopic pipelines produced 244 initial topics from the solitude corpus, which were refined through expert review into 32 high-quality topics using the default pipeline and 46 using the custom pipeline. Similarly, for the gerotranscendence corpus, 172 initial topics were generated, later distilled into 32 and 33 high-quality topics via the default and custom pipelines, respectively. Manual curation across both domains led to a final list of 37 key terms. After filtering the duplicates from both the pipelines, for the PHASES ontology, the BERTopic pipelines contributed a total of 90 terms, 52 from the solitude corpus and 38 from the gerotranscendence corpus. A complete list of these topics is provided in Appendix B.

BERTopic modeling, especially when combined with expert review, can effectively support and speed up the early stages of building an ontology by automatically identifying relevant concepts from a large amount of text. While BERTopic successfully captured many broad themes, such as loneliness, shyness, solitude and loneliness that overlap with SME identified terms, the manually curated list included theory informed concepts like social withdrawal, elective solitude, imposed solitude, ambient sociability, social detachment and so on. These theoretical concepts often require deeper contextual interpretation that automated models may fail to uncover. Nonetheless, the BERTopic model outputs aligned with many foundational ideas, demonstrating its value in generating key concepts for expert refinement. The integration of both algorithmic and expert-driven insights highlighted conceptual overlaps between solitude and gerotranscendence, enriching the semantic foundation for the PHASES ontology.

### 4.4. Merging the results for PHASES ontology development

From the topic modeling outputs containing numbered topics with labels, keywords, and summaries representing the salient content within each domain, to explore thematic connections between solitude and gerotranscendence within the context of healthy aging, a structured text processing pipeline was developed. The analysis began with topic modeling outputs generated by BERTopic modeling for solitude and gerotranscendence, two psychological constructs which have been traditionally studied separately but are relevant for understanding variability in healthy aging. A custom parsing function was implemented to extract and organize the topic data from the BERTopic text files. Using regular expressions, the script identified topic headers and captured their components, including the topic identifier, label, keyword list, and summary. This structured representation allowed for subsequent analysis and filtering of topics based on semantic content. To operationalize the theme of healthy aging, a curated set of keywords was defined, including terms such as “healthy aging,” “well-being,” “successful aging,” “quality of life,” “longevity,” and related expressions involving mental or physical health. This list served as a thematic filter to identify topics that potentially addressed or referenced dimensions of healthy aging. Each topic’s keywords and summary were concatenated and scanned for the presence of any of the predefined healthy aging terms. Topics from both solitude and gerotranscendence models that matched the criteria were retained as relevant. These filtered topic sets were then cross-compared, producing all possible pairwise combinations between solitude-related and gerotranscendence-related topics that were associated with healthy aging. For each identified topic pair, the system recorded topic identifiers, labels, keywords, summaries, and annotated them with a common relationship tag indicating that they were “related via healthy aging theme.” The resulting relations were compiled into a structured dataset and exported as a CSV file to facilitate further analysis and interpretation. This methodology enabled systematic identification of conceptually related topics across two thematic domains, grounded in a consistent and interpretable notion of healthy aging. It provided a reproducible framework for linking topic modeling results to broader theoretical constructs.

Part of the extracted topic relationships between the solitude and gerotranscendence domains as shown in the Table 5 reveal several high-confidence pairings that converge thematically around the experience and interpretation of solitude, as well as its relationship to constructs such as loneliness, aging, and existential crises. Each relationship was identified based on semantic similarity and shared textual context drawn from topic keywords and summaries, with similarity scores ranging from approximately 0.60 to 0.73. Extracted relations include verb-based relational cues such as “is,” “correlates with,” “shows,” “reduces,” and “redefines,” which serve to clarify the nature of the conceptual links between topics. A notable pattern emerges around Topic 3 (“Aloneness”) from the solitude domain, which connects consistently and with relatively high similarity to multiple gerotranscendence topics also labeled “Solitude” (e.g., Topics 6, 76, 92, 123, 162), with similarity scores reaching as high as 0.727. The extracted relations for these pairings suggest recurring themes such as the correlational significance of solitude in the context of aging and well-being (e.g., “correlated negatively with,” “is evident in,” “reduces”), and its redefinition or reinterpretation in later life (e.g., “redefines importance,” “becomes”). Topic 5 (“Solitude”) also maps onto gerotranscendence topics with the same or similar labels, particularly emphasizing its operationalization and measurable correlations. Phrases like “been operationalized as,” “correlates with,” and “need for” indicate a more theoretical or analytical treatment of solitude, possibly reflecting its evolving conceptualization in developmental aging theory. Several additional patterns like in topic 20 (“Solitude”) displays a broader set of connections not only to solitude topics but also to gerotranscendence topics labeled “Aging” (Topic 138) and “Crises” (Topic 169). These links suggest a multifaceted interpretation of solitude that extends beyond peaceful withdrawal to include associations with psychological adaptation, personal transformation, or challenge. The presence of relations like “becomes,” “typically experiences,” and “seem experienced” indicates dynamic, context-dependent meanings of solitude in late life. Topic 41 (“Loneliness”) links internally with topic 33 (“Loneliness”) in the gerotranscendence domain with a strong similarity (0.682), illustrating the nuanced overlap between solitude and loneliness. The extracted relational verbs (e.g., “lacks,” “feel,” “is with”) highlight affective and existential dimensions of this state, reinforcing the need to differentiate loneliness from solitude in ontological modeling. Finally, the appearance of topics such as “Conclusion” and “Conclusions” (Topics 8 and 15) with a high similarity (0.713) suggests a textual match likely due to structural or editorial features rather than conceptual overlap. These are useful reminders of the importance of filtering meta-content in topic modeling pipelines. Overall, this relationship matrix reinforces the theoretical overlap between solitude and gerotranscendence, particularly through the lens of healthy aging. The recurring and thematically rich links around solitude suggest that it operates as a conceptual bridge between internal psychological states and broader existential or developmental processes in later life. The extracted relations provide a valuable linguistic trace of how these connections are articulated in the source texts, which can be formally encoded into the PHASES ontology as candidate object properties or axioms.

**Figure 7:**
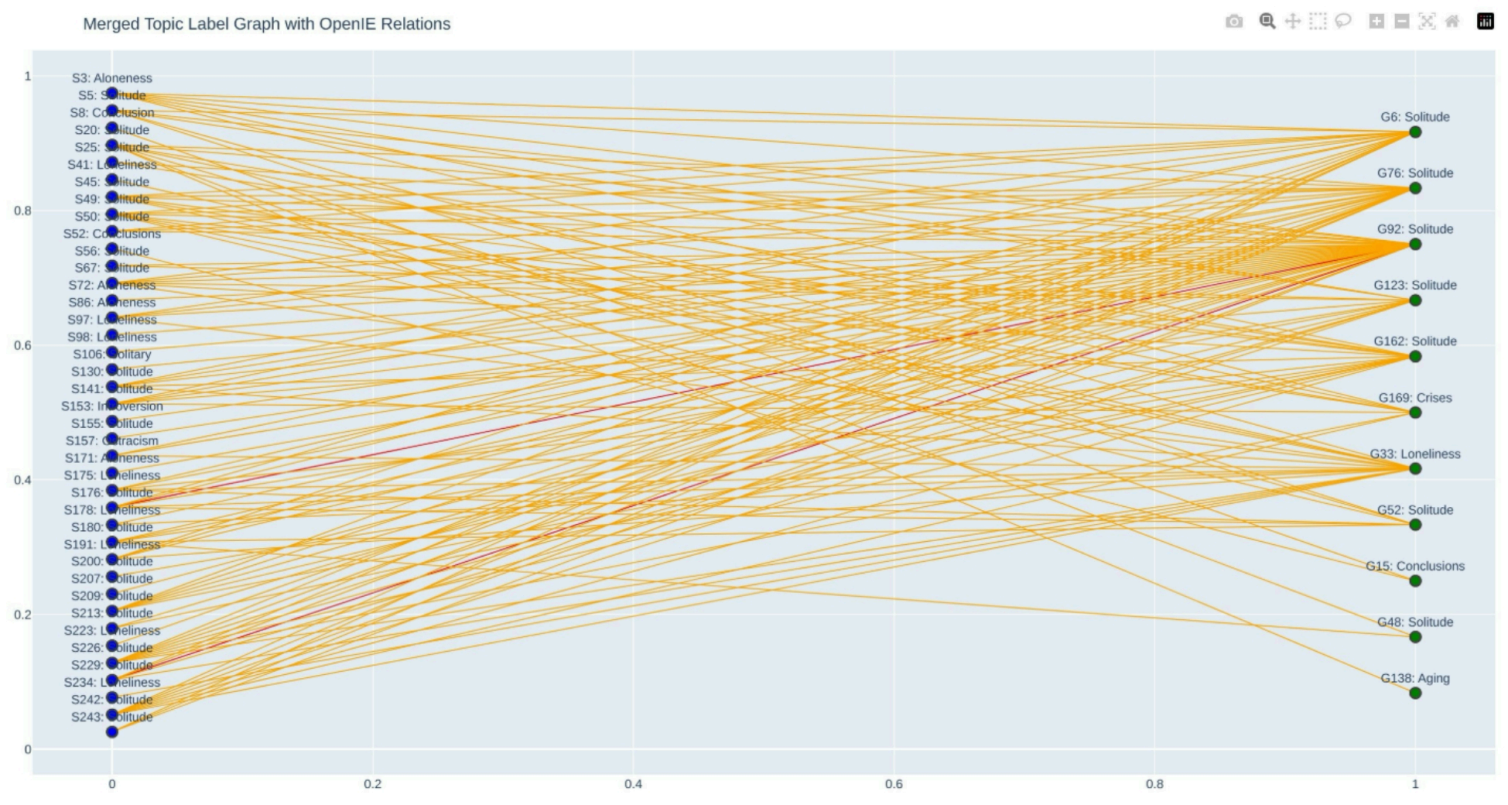
Merged topic label graph with OpenIE relations

The interactive graph in the Fig. 7, generated from the Table 5, visually maps the semantic relationships between solitude and gerotranscendence topics identified through similarity analysis. Each topic’s label, keywords, and summary were concatenated into a single text string and encoded using the all-MiniLM-L6-v2 SentenceTransformer model, after which cosine similarity was computed between all solitude–gerotranscendence pairs. In this pipeline, a cosine similarity threshold of 0.60 was chosen to identify topic pairs that are conceptually related but not necessarily near duplicates. With the all-MiniLM-L6-v2 model, unrelated text pairs typically score around 0.15–0.35, while thematically related pairs often fall between 0.50 and 0.70. Because each topic representation concatenates the label, keywords, and summary, lexical overlap is naturally higher than in pure sentence-to-sentence comparisons; therefore, setting the lower cutoff at 0.60 filters out most chance overlaps while retaining cross-corpus topical neighbors that share domain vocabulary. In contrast, the higher threshold of 0.75 was used to flag “strong matches,” where the labels, keyword sets, and summaries align closely, indicating a high degree of semantic congruence. Scores above this level in “MiniLM” space generally reflect nearly equivalent concepts, even if phrasing differs, making them suitable for red high confidence edges in the visualization. This two-tier approach preserves recall for exploratory connections while distinguishing the most semantically coherent topic alignments.

In the graph, blue nodes represent solitude topics and green nodes represent gerotranscendence topics, arranged in two vertical columns to make cross-domain links visually clear. Red edges indicate strong matches (similarity > 0.75), while orange edges denote related topics (0.60–0.75). Although some labels appear similar or even identical, the associated summaries reveal that each represents a distinctive perspective, highlighting different nuances, contexts, or implications. The layout facilitates immediate recognition of high confidence connections as well as looser thematic links, supporting both targeted analysis and broader exploration. Interactive features allow users to hover over nodes to view full labels and trace their connected edges, enabling direct cross-referencing with the CSV table for keywords, summaries, and extracted OpenIE relations.

## 5. Discussion

This study applied a comparative topic modeling approach using BERTopic to analyze solitude and gerotranscendence corpora, with the overarching goal of facilitating ontology development for the PHASES framework on healthy aging. Both the default BERTopic model and a customized preprocessing pipeline were applied to extract, compare, and interpret meaningful topic structures across corpora. The integration of BART summarization, KeyBERT labeling, and visual analytics (e.g., bar charts, dendrograms) supported both analytical depth and interpretive clarity.

### 5.1 Comparative Performance of Default and Custom BERTopic Pipelines

In both domains, the default BERTopic pipeline yielded broader, more generalizable topic clusters, making it particularly effective for exploratory or high-level thematic mapping. Its outputs displayed clear and interpretable clusters, though at times they suffered from superficial or repetitive keywords, and limited domain sensitivity. Nonetheless, the default approach captured key constructs such as solitude in creative spaces, social exclusion, and disengagement theory providing a useful foundation for downstream analyses. Conversely, the custom preprocessing pipeline significantly enhanced topic granularity and domain relevance. In the solitude corpus, this approach yielded topics such as positive shyness, institutional confinement, and reflective mentorship narratives, concepts that were not as distinct in the default output. For gerotranscendence, custom preprocessing uncovered intricate subthemes such as cosmic gendered experiences, behavioral manifestations of transcendence, and solitary meaning-making in later life. Despite occasional keyword redundancy, the custom method offered superior resolution for identifying and interpreting nuanced psychological and developmental constructs. Both pipelines leveraged class-based TF-IDF (c-TF-IDF) for topic representation, but custom preprocessing enhanced this step through domain-sensitive feature selection, including POS tagging and phrase filtering. While the default pipeline benefited from minimal preprocessing that preserved general word frequency patterns, the custom approach foregrounded psychological, existential, and narrative terms that are central to solitude and gerotranscendence discourses.

Across both corpora, solitude emerged not merely as physical aloneness, but as a multifaceted psychological and developmental phenomenon. Themes ranged from enforced isolation (e.g., solitary confinement) and social ostracism to creative solitude, philosophical inquiry, and personality traits like shyness. The custom pipeline especially surfaced solitude’s conceptual evolution—from pathological states to deliberate, enriching experiences tied to reflection, growth, and resilience. In the gerotranscendence corpus, topic modeling revealed a similarly layered structure. Topics included theoretical foundations (e.g., disengagement theory), gendered or spiritual transitions, caregiving dynamics, and the cognitive-emotional reorientation of aging individuals. The intersection of solitude and gerotranscendence became particularly salient through topics that addressed positive solitude, social withdrawal, identity shifts, and existential reinterpretations of aging. The BART-generated summaries were instrumental in deepening interpretation. They bridged the gap between keyword-based topics and human-understandable narratives, allowing for richer insights into how solitude and aging are framed across empirical, theoretical, and lived experience contexts.

### 5.2 Ontology Development and Thematic Integration

The integration of topic modeling outputs into the PHASES ontology framework represents a key methodological contribution. By filtering topics based on relevance to “healthy aging” operationalized via curated terms such as well-being, successful aging, and quality of life, the study identified conceptually rich topic pairs spanning both domains. These were semantically matched and annotated using relational cues (e.g., correlates with, reduces, redefines), enabling the construction of a structured, interpretable map of cross-domain relationships. High-confidence pairings, such as the repeated connections between “Aloneness” in solitude and “Solitude” in gerotranscendence, reinforced solitude’s centrality as a psychological and existential construct in later life. Topic links involving “Loneliness” and “Crises” further illustrated how affective and transitional experiences contribute to aging narratives. Additionally, the detection of meta-topics such as “Conclusion” emphasized the need for filtering structural artifacts during topic interpretation. This structured approach to relationship extraction laid the groundwork for encoding meaningful semantic relations into the PHASES ontology. These include candidate object properties (e.g., redefines, correlates with) and potential class axioms, providing a foundation for automated reasoning and conceptual modeling in future knowledge engineering efforts.

## 6. Conclusion

This study employed a dual-pipeline BERTopic strategy to map overlapping and domain-specific themes in solitude and gerotranscendence literature, highlighting their conceptual interconnectedness within the broader discourse on healthy aging. The approach addressed a key limitation of traditional ontology development, its heavy reliance on subject matter experts (SMEs), high time demands, and vulnerability to cognitive bias by introducing a semi-automated, workflow for early-stage concept extraction. Applied to The Handbook of Solitude and Gerotranscendence, the pipelines generated 416 initial topics, refined through SME review to 32 (custom) and 46 (default) high quality solitude topics, and 33 (custom) and 32 (default) high-quality gerotranscendence topics, yielding 90 ontology terms in total (52 from solitude, 38 from gerotranscendence).

While both BERTopic pipelines have their strengths, the custom preprocessing version offers superior specificity and interpretive granularity, whereas the default excels in abstraction and structural coherence, beneficial for building high-level thematic maps. The custom pipeline’s richer multi-word expressions and removal of low-value tokens produce more coherent topics, enhancing interpretability for domain-specific research. The default pipeline, in contrast, provides broader exploratory coverage, though it risks more noise. Visual evaluations confirmed these differences, with the custom pipeline excelling in interpretive granularity. Future improvements to the custom approach could include post-vectorization de-duplication, filtering of citation patterns, and merging of semantically identical n-grams to ensure each topic’s top terms are both unique and highly informative. Certain theory-heavy concepts still required SME interpretation, underscoring the complementary role of automated methods and expert review. By systematically mapping the interrelations between solitude and gerotranscendence, this work not only contributes to empirical understanding in psychology and gerontology but also offers a reproducible framework for integrating unsupervised NLP outputs into structured ontologies. These findings may inform future interventions, policy discussions, and theoretical explorations around aging, well-being, and the transformative role of solitude across the human lifespan.

To integrate outputs across the solitude and gerotranscendence domains, semantic embeddings of topic labels, keywords, and summaries from the custom pipeline were compared using cosine similarity, identifying semantically matched topic pairs above a set threshold. This enabled the construction of a merged conceptual framework that bridges both domains and strengthens the semantic foundation of the PHASES Ontology.

From the findings of our study, several areas for future research can be explored. Some of them are:

- Automatic generation of descriptions, definitions, and relationships between the keywords for ontology development using LLMs (Large Language Models).
- Competency questions (CQs) guide what an ontology should include and give a better idea of what an ontological domain would be. Generating competency questions from keywords can supplement the competency questions generated by experts.
- Further incorporation of more powerful LLMs (e.g., ChatGPT).

BERTopic Modeling Implementation, along with supporting documentation, can be found here: https://github.com/Buffalo-Ontology-Group/phases-nlp

## Supplementary Material

This is a list of supplementary files associated with this preprint. Click to download.

• Appendices.docx

## Figures and Tables

**Table 1:** BART-generated topic summaries for the default BERTopic model applied to the solitude corpus.

Topic	Topic Label	Keywords	Summary
0	Solitary	confinement, prisoners, solitary, prison, isolation	Solitary confinement is a form of solitary confinement in which prisoners are isolated from the rest of the prison population. Solitary confinement is so common in prisons that it has been dubbed a “socially accepted’ practice.
1	Creativity	creative, ideas, creativity, efforts, paulus	Creative people tend to have some characteristics in common with “lone geniuses,” such as broad interests, and independence. But the key factors in many cases is simple hard work, persistence, and a resultant high quantity of creative products.
2	Singlehood	singlehood, single, perspective, depaulo, reasons	Singlehood is a phenomenon that is determined by multiple factors involving social, cultural, religious, and psychological ones. This chapter will present the current knowledge on singlehood from a psychological perspective.
3	Aloneness	solitude, chapters, paradox, psychoanalytic, perspectives	The Handbook of Solitude was the first academic volume to specifically focus on the diverse theoretical and empirical approaches to the psychological study of solitude. In this second edition of the Handbook, we are absolutely thrilled to present a blend of new and updated chapters that approach solitude from a myriad of theoretical perspectives and methodological approaches.
4	Ostracism	ostracism, williams, chronic, individuals, ignored	Ostracism – being ignored and excluded – is a social phenomenon that occurs in a myriad cultures and contexts among humans and nonhuman social animals. Evolutionary theory offers an explanation for the robustness of ostracism in human society and its occurrence within groups of other social animals.
5	Solitude	presence, definition, alone, physically, solitude	Solitude can be and sometimes has been operationalized as an absence of social interaction and sometimes as physical aloneness. There is really no agreed-upon physical “distance” from others that is mandated in order for an individual to be considered alone.

**Table 2:** BART-generated topic summaries for the custom-preprocessed BERTopic model applied to the solitude corpus.

Topic	Topic Label	Keywords	Summary
0	Solitary	confinement, solitary confinement, prisoners, prison, solitary	Solitary confinement is a form of solitary confinement in which prisoners are isolated from the rest of the prison population. Solitary confinement is so common in prisons that it has been dubbed a “socially accepted’ practice.
1	Isolation	ideas, creative, creativity, efforts, creative process	Creative people tend to have some characteristics in common with “lone geniuses,” such as broad interests, and independence. But the key factors in many cases is simple hard work, persistence, and a resultant high quantity of creative products.
2	Single	singlehood, single, single people, mating, żurek	Singlehood is a phenomenon that is determined by multiple factors involving social, cultural, religious, and psychological ones. This chapter will present the current knowledge on singlehood from a psychological perspective.
3	Aloneness	solitude, chapters, paradox, psychoanalytic, beneficial	The Handbook of Solitude was the first academic volume to specifically focus on the diverse theoretical and empirical approaches to the psychological study of solitude. In this second edition of the Handbook, we are absolutely thrilled to present a blend of new and updated chapters that approach solitude from a myriad of theoretical perspectives and methodological approaches.
4	Ostracism	ostracism, chronic ostracism, chronic, effects ostracism, ignored	Ostracism – being ignored and excluded – is a social phenomenon that occurs in a myriad cultures and contexts among humans and nonhuman social animals. Evolutionary theory offers an explanation for the robustness of ostracism in human society and its occurrence within groups of other social animals.
5	Solitude	definition, presence, definition solitude, physically, café	Solitude can be and sometimes has been operationalized as an absence of social interaction and sometimes as physical aloneness. There is really no agreed-upon physical “distance” from others that is mandated in order for an individual to be considered alone.

**Table 3:** BART-generated topic summaries for the default BERTopic model applied to the gerotranscendence corpus.

Topic	Topic Label	Keywords	Summary
0	Gerontology	theories, gerontology, theoretical, gerontological, departure	Theory of gerotranscendence was developed from unsatisfying mismatch of common theoretical assumptions within social gerontology and some empirical findings. We will argue that the usual theoretical points of departure for gerontological research only represent a narrow corridor in a theoretical field, which is much broader.
1	Nursing	staff, nurses, percent, areas, impact	Familiarity with the Theory Seemed to Reduce Feelings of Insufficiency at Work. Theory Helped Many Staff Members to See Care Recipients in a New Light and to Understand Them Better.
2	Dissatisfaction	disengagement, theory, cumming, individual, hypothesis	Cumming, Newell, Dean, & McCaffrey (1960) first published their tentative disengagement theory of aging. The theory assumed an intrinsic tendency to disengage and withdraw when growing old.
3	Gerotranscendence	gerotranscendence, practice, towards, acceler, tenth	Gerotranscendence can be facilitated or obstructed in different ways. The process of gerotrans transcendence can, according to the theory, be accelerated, facilitated or Obstructed.
4	Depression	interviewees, sign, isolation, elderly, pleasure	A lack of interest in participating in parties can be understood as a symptom of a beginning dementia (the pathology perspective) or as a way to cope with reduced mobility by means of selecting where and how often to go to parties (the SOC perspective) Robinson, 1976; Bradford, 1979; Bernard, 1982. Other indications came from subjective reports of staff members working with old people.
5	Cosmic	women, men, cosmic, score, category	Women score higher than men on cosmic transcendence, but that this difference decreases with increasing age. The women’s higher levels of cosmic transcendence, especially in the age interval 25–44, might be partly connected with childbirth.

**Table 4:** BART-generated topic summaries for the custom-preprocessed BERTopic model applied to the gerotranscendence corpus.

Topic	Topic Label	Keywords	Summary
0	Definitions	gerontology, theories, points departure, gerontological, departure	Theory of gerotranscendence was developed from unsatisfying mismatch of common theoretical assumptions within social gerontology and some empirical findings. We will argue that the usual theoretical points of departure for gerontological research only represent a narrow corridor in a theoretical field, which is much broader.
1	Nursing	staff, nurses, areas impact, percent, areas	Familiarity with the Theory Seemed to Reduce Feelings of Insufficiency at Work. Theory Helped Many Staff Members to See Care Recipients in a New Light and to Understand Them Better.
2	Dissatisfaction	disengagement, disengagement theory, cumming, theory, disengage	Cumming, Newell, Dean, & McCaffrey (1960) first published their tentative disengagement theory of aging. The theory assumed an intrinsic tendency to disengage and withdraw when growing old.
3	Gerotranscendence	practice gerotranscendence, gerotranscendence practice gerotranscendence, gerotranscendence practice, gerotranscendence, practice	Gerotranscendence can be facilitated or obstructed in different ways. The process of gerotrans transcendence can, according to the theory, be accelerated, facilitated or Obstructed.
4	Depression	interviewees, sign, isolation, parties, elderly	A lack of interest in participating in parties can be understood as a symptom of a beginning dementia (the pathology perspective) or as a way to cope with reduced mobility by means of selecting where and how often to go to parties (the SOC perspective) Robinson, 1976; Bradford, 1979; Bernard, 1982. Other indications came from subjective reports of staff members working with old people.
5	Transcendence	women, men, cosmic transcendence, cosmic, women score higher	Women score higher than men on cosmic transcendence, but that this difference decreases with increasing age. The women’s higher levels of cosmic transcendence, especially in the age interval 25–44, might be partly connected with childbirth.

**Table 5:** Part of topic pairings between solitude and gerotranscendence

solitude_id	solitude_label	gerotranscendence_id	gerotranscendence_label	similarity	relationship	merged_topic	extracted_relations
3	Aloneness	6	Solitude	0.6496	related	Aloneness - Solitude	‘are absolutely thrilled in’, ‘present’, ‘is highly evident in’, ‘is evident in’
3	Aloneness	169	Crises	0.637	related	Aloneness - Crises	‘are absolutely thrilled in’, ‘present’, ‘seem In’, ‘experienced’
5	Solitude	33	Loneliness	0.633	related	Solitude - Loneliness	‘sometimes been operationalized as’, ‘lacks’, ‘may’, ‘be further’
20	Solitude	138	Aging	0.607	related	Solitude - Aging	‘is in’, ‘typically experiences’
45	Solitude	169	Crises	0.608	related	Solitude - Crises	‘presents’, ‘is in’, seem In’, ‘experienced’

## Abbreviations

SME: Subject Matter Experts
NLP: Natural Language Processing
LLM: Large Language Models
RAG: Retrieval-Augmented Generation
PHASES: Promoting Healthy Aging through Semantic Enrichment of Solitude Research
NER: Named Entity Recognition
SHAP: SHapley Additive exPlanations
HRQL: Health-Related Quality of Life
LDA: Latent Dirichlet Allocation
OWL: Web Ontology Language
RIGOR: Retrieval-Augmented Iterative Generation framework
OE: Ontology Engineering
NMF: Non-negative Matrix Factorization
LSA: Latent Semantic Analysis
UMAP: Uniform Manifold Approximation and Projection
HDBSCAN: Hierarchical Density-Based Spatial Clustering of Applications with Noise
c-TF-IDF: class-based Term Frequency - Inverse Document Frequency
AUROC: Area Under the Receiver Operating Characteristic
CQ: Competency Questions
TF-IDF: Term Frequency - Inverse Document Frequency
BART: Bidirectional and Auto-Regressive Transformer
POS: Part-Of-Speech
